# Identification of Perioperative Procedural and Hemodynamic Risk Factors for Developing Colonic Ischemia after Ruptured Infrarenal Abdominal Aortic Aneurysm Surgery: A Single-Centre Retrospective Cohort Study

**DOI:** 10.3390/jcm12124159

**Published:** 2023-06-20

**Authors:** Safwan Omran, Larissa Schawe, Frank Konietschke, Stefan Angermair, Benjamin Weixler, Sascha Treskatsch, Andreas Greiner, Christian Berger

**Affiliations:** 1 Department of Vascular Surgery, Corporate Member of Freie Universität Berlin and Humboldt-Universität zu Berlin, Charité—Universitätsmedizin Berlin, Hindenburgdamm 30, 12203 Berlin, Germany; larissa.schawe@charite.de (L.S.); andreas.greiner@charite.de (A.G.); 2 Institute of Medical Biometrics and Clinical Epidemiology, Corporate Member of Freie Universität Berlin and Humboldt-Universität zu Berlin, Charité—Universitätsmedizin Berlin, 10117 Berlin, Germany; frank.konietschke@charite.de; 3 Berlin Institute of Health (BIH), Charité—Universitätsmedizin Berlin, 10178 Berlin, Germany; 4 Department of Anesthesiology and Intensive Care Medicine, Charité Campus Benjamin Franklin, Corporate Member of Freie Universität Berlin and Humboldt-Universität zu Berlin, Charité—Universitätsmedizin Berlin, Hindenburgdamm 30, 12203 Berlin, Germany; stefan.angermair@charite.de (S.A.); sascha.treskatsch@charite.de (S.T.); christian.berger@charite.de (C.B.); 5 Department of General and Visceral Surgery, Corporate Member of Freie Universität Berlin and Humboldt-Universität zu Berlin, Charité—Universitätsmedizin Berlin, Hindenburgdamm 30, 12203 Berlin, Germany; benjamin.weixler@charite.de

**Keywords:** ruptured abdominal aortic aneurysm, colonic ischemia, catecholamines, norepinephrine, ischemic colitis

## Abstract

(1) Background: This retrospective study evaluated perioperative and intensive care unit (ICU) variables to predict colonic ischemia (CI) after infrarenal ruptured abdominal aortic aneurysm (RAAA) surgery. (2) Materials and Methods: We retrospectively analyzed the data of the patients treated for infrarenal RAAA from January 2011 to December 2020 in our hospital. (3) Results: A total of 135 (82% male) patients were admitted to ICU after treatment of infrarenal RAAA. The median age of all patients was 75 years (IQR 68–81 years). Of those, 24 (18%) patients developed CI, including 22 (92%) cases within the first three postoperative days. CI was found more often after open repair compared to endovascular treatment (22% vs. 5%, *p* = 0.021). Laboratory findings in the first seven PODs revealed statistically significant differences between CI and non-CI patients for serum lactate, minimum pH, serum bicarbonate, and platelet count. Norepinephrine (NE) was used in 92 (68%) patients during ICU stay. The highest daily dose of norepinephrine was administered to CI patients at POD1. Multivariable analysis revealed that NE > 64 µg/kg (RD 0.40, 95% CI: 0.25–0.55, *p* < 0.001), operating time ≥ 200 min (RD 0.18, 95% CI: 0.05–0.31, *p* = 0.042), and pH < 7.3 (RD 0.21, 95% CI: 0.07–0.35, *p* = 0.019), significantly predicted the development of CI. A total of 23 (17%) patients died during the hospital stay, including 8 (33%) patients from the CI group and 15 (7%) from the non-CI group (*p* = 0.032). (4) Conclusions: CI after RAAA is a sever complication occurring most frequently within the first 3 postoperative days. Our study identified many surrogate markers associated with colonic ischemia after aortic RAAA, including norepinephrine dose > 64 µg/kg, operating time ≥ 200 min, and PH < 7.3. Future studies are needed to support these results.

## 1. Introduction

Colonic Ischemia (CI) is a well-known complication of aortic surgery with potentially high morbidity and mortality rates. Recent studies have shown a CI incidence of 0.5–3% after elective abdominal aortic aneurysm (AAA) surgery [1,2,3,4] with an increased incidence of 10% to 36% after ruptured abdominal aortic aneurysms (RAAA) [4,5,6].

Despite improvements in operative strategies and postoperative care, mortality rates of CI after RAAA repair are still high and have exceeded 60% in some studies [4,5,6]. Most publications addressing risk factors for developing CI after AAA surgery have shown that repair of RAAA, in contrast to elective surgery, is an important determining factor [7,8,9,10]. Although many studies reported on risk factors for developing CI after aortic surgery, only a few reported on those after RAAA [9,10,11]. Additionally, many reports about CI focus on patients’ demographic, comorbidities, and operative factors [12]. However, perioperative hemodynamic factors (e.g., hypertension) were scarcely studied in the last decade [6,10].

Therefore, this study aims to report and analyze perioperative procedural and hemodynamic risk factors for developing CI after RAAA surgery.

## 2. Materials and Methods

The ethics committee of the Charité—Universitätsmedizin Berlin approved the present study (Approval date: 27 May 2020, No.: EA4/088/20). Due to its retrospective nature, the ethics committee waived informed consent. This retrospective study included patients who underwent operations on infrarenal RAAA and were afterward admitted to ICU between January 2011 and December 2020 [13]. Patients with ruptured suprarenal, thoracic, or thoracoabdominal aortic aneurysms were excluded. Additionally, all RAAA patients with preoperative or intraoperative deaths were excluded. Patients were divided into two groups: those who developed postoperative colonic ischemia (CI group) and those who did not (non-CI group). Patient characteristics, intraoperative, and ICU data for seven consecutive postoperative days (POD) were collected from two institutional patient databases (COPRA System GmbH, Sasbachwalden, Germany, and SAP AG, Walldorf, Germany).

For day-by-day analysis of the treatment during the ICU stay, postoperative day 0 (POD0) was defined as the calendar day of surgery. Each subsequent complete day was defined and abbreviated as POD1, POD2, etc.

Included in the study were patients who underwent endovascular or open surgical repair of the infrarenal aorta. A 24-h emergency endovascular aortic repair (EVAR) service and endovascular-first strategy have been offered since 2015. The decision to use endovascular or open surgical repair is based on several factors including hemodynamic status, aortic anatomy, availability of suitable endovascular devices, and the operator’s level of expertise. Resuscitative endovascular balloon occlusion of the aorta (REBOA) was not performed routinely in or study. Re-implantation of the inferior mesenteric artery (IMA) and colonic endoscopy after surgery were not performed routinely. Therefore, only patients with a clinical picture suggestive of CI, including rectal bleeding, abdominal distention, diarrhea, elevated lactate, increasing white blood cell counts, and sepsis, were considered for endoscopy or exploratory laparotomy. Additionally, patients who underwent open abdomen therapy to prevent or treat abdominal compartment syndrome were considered for a second look and therefore underwent no endoscopy. The diagnosis of CI was based on either postoperative endoscopy or exploratory laparotomy. Based on surgical and endoscopic findings, CI was classified into three stages: I: transient mucosal ischemia, II: mucosal and muscular involvement, and III: transmural ischemia and infarction [14].

Preoperatively collected and analyzed data included patient demographics, cardiovascular risk factors, comorbidities, computer tomography (CT) findings, and preoperative clinical presentation. Hemodynamic instability was defined as systolic blood pressure < 90 mmHg. Additionally, preoperative cardiopulmonary resuscitation and loss of consciousness were documented. Additionally, we evaluated the preoperative status and postoperative patency of the inferior mesenteric artery and internal iliac (hypogastric) arteries. Intraoperative parameters included duration of operation, suprarenal aortic cross-clamping, and transfusion of packed red blood cells (PRBCs).

Day-wise analysis of the subsequent seven postoperative days of ICU treatment included weight-based doses and types of catecholamines, the amount of postoperative fluid administered, mean arterial pressure (MAP), mean heart rate (HR), minimum pH, minimum serum bicarbonate, minimum hemoglobin, minimum platelet count, maximum serum lactate, mean partial pressure of oxygen (PaO_2_), maximum leucocytes count, maximum C-reactive protein (CRP), and maximum creatinine. The catecholamine doses were calculated in a microgram per kilogram of body weight. A MAP target of 65 mm Hg was deemed adequate for most patients [15,16]. The optimal cutoff points of the variables (norepinephrine, operating time, PRBCs, lactate, and pH) to discriminate patients with CI from those without were assessed using the area under the receiver operating curve (ROC) and the Youden index.

Patients in endoscopically detected stage I (transient mucosal ischemia) underwent non-operative treatment with antibiotic therapy, intravenous fluids, and bowel rest. Additionally, patients in stages II (mucosal and muscular involvement) and III (transmural ischemia) underwent immediate exploratory laparotomy and colonic resection with the avoidance of primary anastomosis. In addition, major perioperative complications, length of stay in the ICU and hospital, in-hospital mortality, and cause of death were analyzed. This study was registered in the German Clinical Trials Registry DRKS—Deutsches Register Klinischer Studien, no. DRKS00031369.

### Statistical Methods

Whenever possible, comparison between groups was performed by calculating the odds ratio (OR), with 95% confidence intervals, whereas OR refers to the patient’s risk of developing CI after ruptured aortic aneurysms surgery. Results are presented as mean ± SD (standard deviation) for symmetrically distributed data, and as median (interquartile range [IQR]) for skewed data. Missing data were not imputed. The missing data came from not all patients being in intensive care unit (ICU) for seven days. In addition, univariate analysis was performed with the χ2 test or Fisher exact test for categorical data and the student *t*-test or Mann–Whitney nonparametric test for numerical data to analyze the risk factors for CI development. The optimal cutoff points to discriminate patients with CI from those without were assessed using a receiver-operating curve (ROC) analysis, the area under the ROC curve (AUC) and the Youden index. Variables were considered significantly associated with CI at a *p* value < 0.20 and were entered into a multivariable model using backward stepwise logistic regression. We checked the main assumptions of logistic regression analysis using correlation analysis to avoid multicollinearity problems. Additionally, we computed the risk difference (RD) for each significant predictor of CI obtained from the multivariable model. The statistical analysis was performed using SPSS Statistics 26 for Windows (IBM, Armonk, NY, USA).

## 3. Results

**Figure 1:**
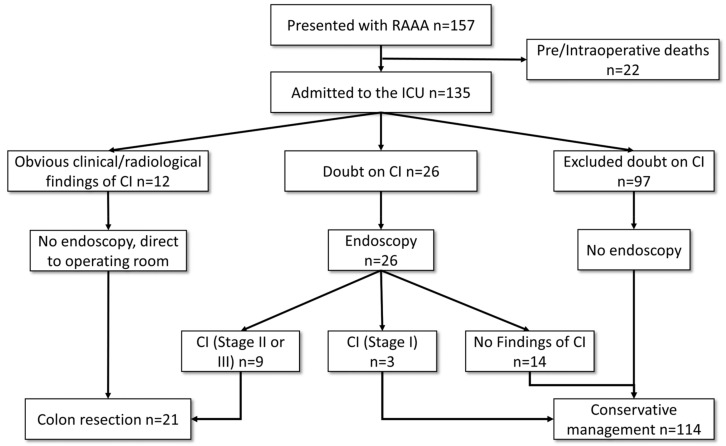
Flowchart of study patients and decision-making.

From January 2011 to December 2020, 157 patients presented with RAAA in our tertiary university center (see Figure 1). A total of 22 patients with preoperative or intraoperative deaths were excluded from this study. As a result, 135 patients who underwent surgical treatment and were subsequently admitted to the ICU were included. Of those, 24 (18%) patients developed CI postoperatively (CI group), and 111 (82%) patients did not (non-CI group). The median age of all patients was 75 years (IQR 68–81 years), and 111 (82%) were male. Patients’ demographics and medical history showed no difference between the two groups except for coronary artery disease (CAD), which was higher in the CI group (38% vs. 17%, *p* = 0.026) (Table 1).

Twenty-six (19%) patients underwent colonoscopy due to suspected CI. Of those, twelve showed signs of colon ischemia resulting in subsequent colonic resection (see Figure 1). Additionally, 12 CI patients underwent no endoscopy because they had obvious clinical and radiological findings of CI; therefore, a second look to explore the colon was performed.

Five (21%) patients developed CI within POD0, five (21%) on POD1, ten (42%) on POD2, two (8%) on POD3, one (4%) on POD8, and one (4%) on POD12. Accordingly, 22 (92%) patients of the CI group developed CI in the first 3 postoperative days. Patients of the CI group were subdivided into three stages of ischemia based on the surgical and endoscopic findings: stage I occurred in 3 (13%) patients, stage II in 2 (8%), and stage III in 19 (80%) patients. CI occurred more often after open repair (22/98, 22%) compared to endovascular treatment (2/37, 5%) (*p* = 0.021). Ten (7%) patients underwent open abdomen therapy to prevent or treat abdominal compartment syndrome.

**Figure 2:**
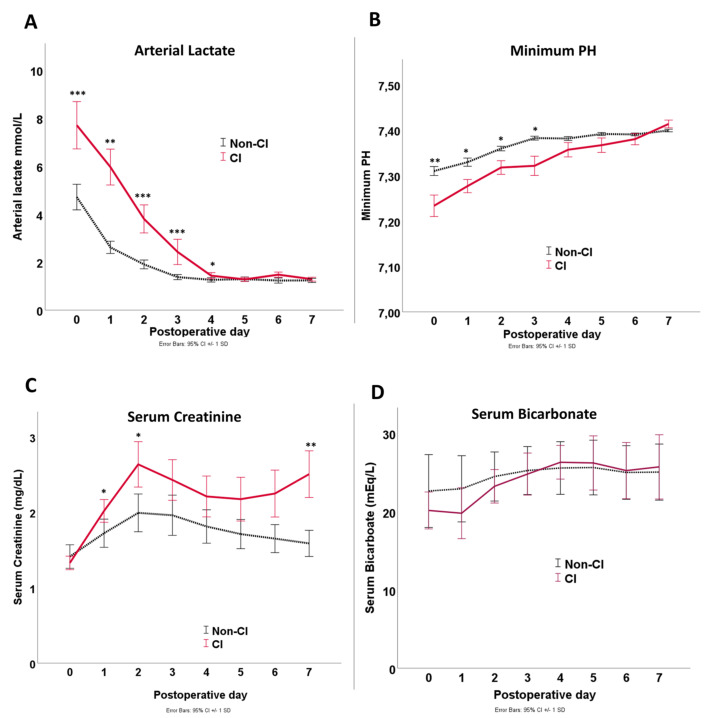
Line graph of laboratory findings for arterial lactate (**A**), minimum pH (**B**), serum creatinine (**C**), and serum bicarbonate (**D**) during the first seven postoperative days after RAAA aortic repair. Error bars indicate standard deviation. Asterisks indicate significant differences: * *p* < 0.05; ** *p* < 0.01; *** *p* < 0.001. *p*-values are based on *t*-tests.

**Figure 3:**
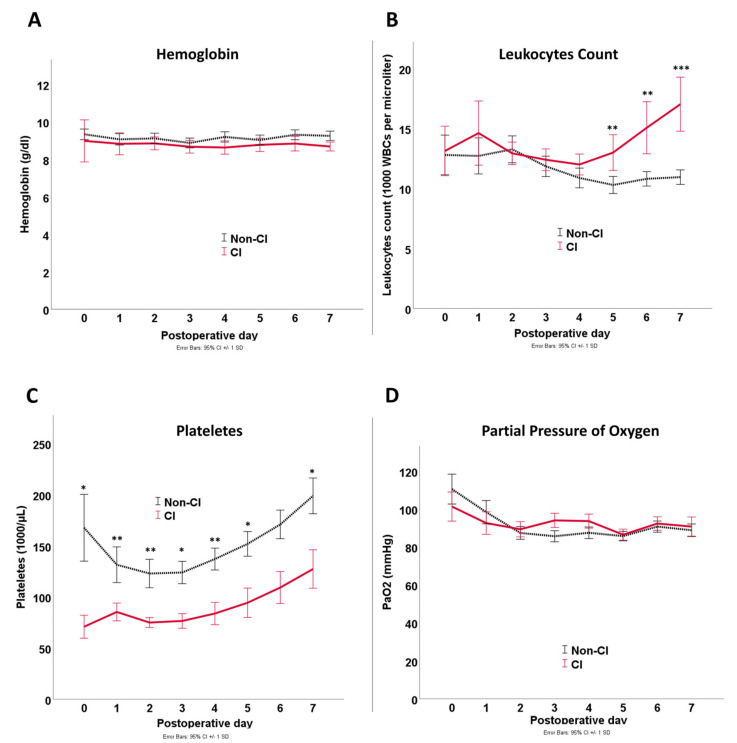
Line graph of laboratory findings for hemoglobin (**A**), leucocytes count (**B**), platelets (**C**), and PaO_2_ (**D**) during the first seven postoperative days after RAAA aortic repair. Error bars indicate standard deviation. Asterisks indicate significant differences: * *p* < 0.05; ** *p* < 0.01; *** *p* < 0.001. *p*-values are based on *t*-tests.

Laboratory findings in the first seven days are depicted in Figure 2 and Figure 3, which revealed statistically significant differences between CI and non-CI patients for serum lactate, minimum pH, serum bicarbonate, and platelet count. Hemoglobin and PaO_2_ showed no statistically significant differences between both groups at any time point. On the other hand, leukocytes showed higher values in the CI group from POD5 and serum creatinine from POD2 onwards.

**Figure 4:**
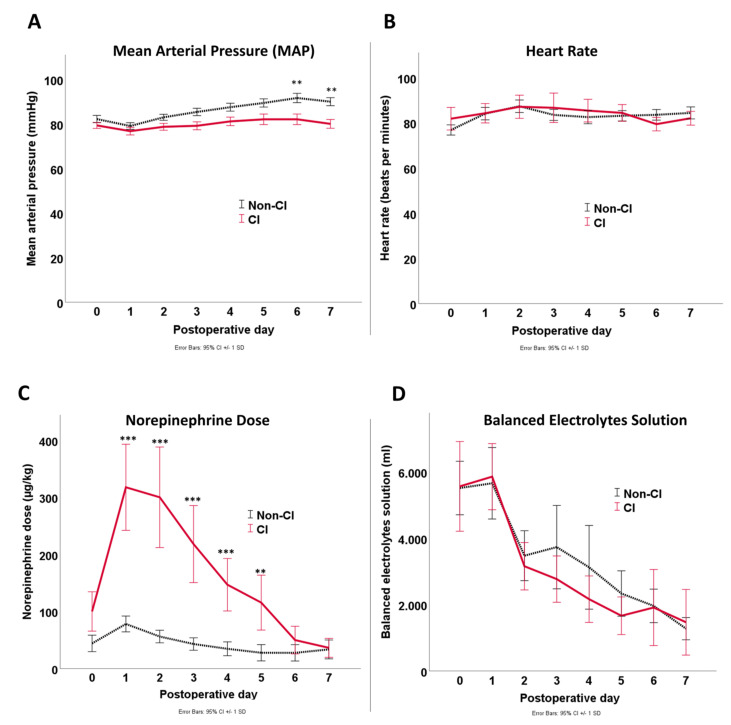
Line graph of the mean arterial pressure (**A**), heart rate (**B**), norepinephrine dose (**C**), and fluid (balanced electrolytes solutions) treatment (**D**) during the first the first seven postoperative days after RAAA aortic repair. Error bars in-dicate standard deviation. Asterisks indicate significant differences: * *p* < 0.05; ** *p* < 0.01; *** *p* < 0.001. *p*-values are based on *t*-tests.

Hemodynamic variables within the first week of ICU therapy are presented in Figure 4. The MAP target of at least 65 mmHg was achieved in both groups. However, MAP was higher in the non-CI group at some time points. This MAP was achieved with a comparable heart rate (HR) and a cumulative amount of fluids administered in both groups but with significantly higher norepinephrine dosages in the CI group.

Norepinephrine (NE) was the most frequently used catecholamine during ICU in 92 (68%) patients. In total, 24 (100%) patients in CI group received NE and 68 (61%) in non-CI group. Other catecholamines included dobutamine in 14 (10%) and, epinephrine in 7 (5%) patients. Further administered non-catecholamine inotropes or vasopressors were enoximone in nine (7%) and vasopressin (inclusive analogs) in five (4%) patients of all cases.

The necessity for catecholamines to maintain the hemodynamic stability was significantly higher in the CI group (100% vs. 70%, *p* = 0.002). Additionally, 18 (13%) patients needed two or more catecholamines, including 7 (29%) patients in the CI group and 11 (10%) patients in the non-CI group (*p* = 0.020).

**Figure 5:**
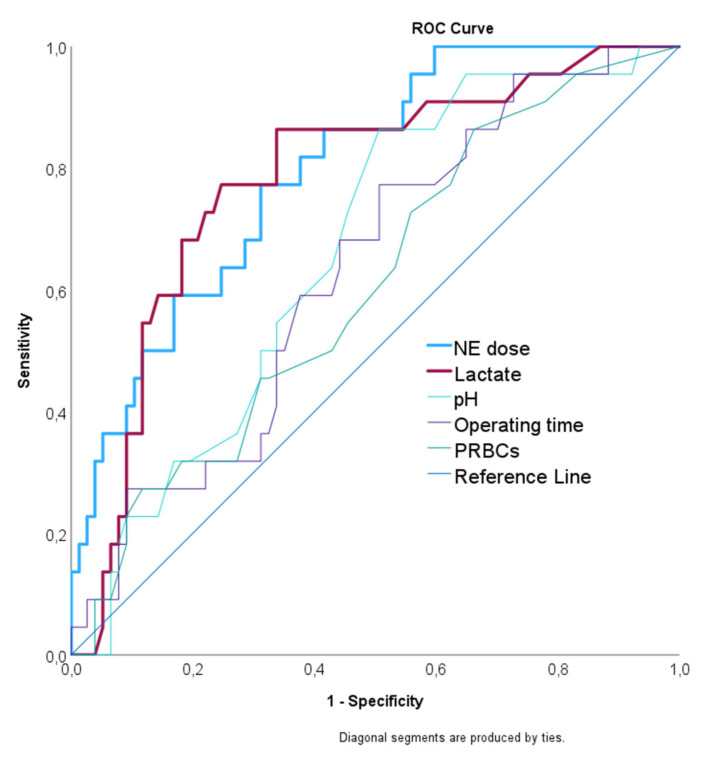
Receiver operating characteristic curves for the norepinephrine (NE) dose on POD1, maximum serum lactate on POD1, packed red blood cells (PRBCs), operating time, and minimum pH on POD1.

The highest daily NE dose was administered to CI patients at POD1 (Figure 4c). The area under the ROC curve of NE dose on POD1 for predicting CI incidence was 0.80 (CI: 0.70–0.89, *p* < 001) with a best cut-off point of 64 µg/kg/day. Similarly, the AUC for maximum serum lactate was 0.78 (CI: 0.67–0.89, *p* < 0.001) with a best cut-off point of 5 mmol/L. The AUC for packed red blood cells (PRBCs) was 0.61 (CI: 0.48–0.73, *p* = 0.134) with a best cut-off point of 5 units. The AUC for operating time was 0.63 (CI: 0.51–0.76, *p* = 0.061) with a best cut-off point of 200 min. Finally, the AUC for pH was 0.71 (CI: 0.61–0.82, *p* = 0.020) with a best cut-off point of 7.3. Therefore, the optimal cut-off points for operating time (200 min), PRBCs (5 units), pH (7.3), and lactate (5 mmol/L) were used to discriminate between CI and non-CI groups (Figure 5).

Analysis of those parameters and risk factors of CI after RAAA repair by univariate logistic regression is depicted in Table 2. Statistically significant predictors for CI in the univariate analysis included preoperative coronary artery disease (OR 2.9, 95% CI: 1.1–7.6, *p* = 0.026), hemodynamic instability (OR 4.9, 95% CI: 1.9–12.7, *p* = 0.001), cardiopulmonary resuscitation (OR 7.0, 95% CI: 2.0–25.6, *p* = 0.004), and loss of consciousness (OR 3.9, 95% CI: 1.5–10.3, *p* = 0.009). Intraoperative statistically significant predictors were open aortic repair (OR 5.1, 95% CI: 1.1–22.7, *p* = 0.021), transfusion of ≥ 5 units PRBCs (OR 3.3, 95% CI: 1.2–8.9, *p* = 0.015), and operating time ≥ 200 min (OR 3.8, 95% CI: 1.4–10.3, *p* =.006). Additionally, pH < 7.3 (OR 4.5, 95% CI: 1.7–11.7, *p* = 0.001), NE > 64 µg/kg (OR 17.2, 95% CI: 5.4–55.0, *p* < 0.001), and arterial lactate > 5 mmol/L (OR 10.3, 95% CI: 3.5–29.9, *p* < 0.001) showed statistically significant differences at POD1 between both groups.

Significant risk factors with a *p*-value < 0.05 in the univariable analysis were thus included in a logistic regression model using backward selection to predict CI. We checked the main assumptions of logistic regression analysis using correlation analysis to avoid multicollinearity problems. The variables included in the models were not highly correlated; that is, no correlation was greater than 0.7. Multivariable analysis revealed that NE > 64 µg/kg [risk difference (RD) 0.40, 95% CI: 0.25–0.55, *p* < 0.001], operating time ≥ 200 min (RD 0.18, 95% CI: 0.05–0.31, *p* = 0.042), and PH < 7.3 (RD 0.21, 95% CI: 0.07–0.35, *p* = 0.019) significantly predicted the development of CI. The Hosmer–Lemeshow goodness-of-fit test statistic (chi-square) was 8.3 with a *p*-value of 0.14.

Overall postoperative complications revealed no statistically significant difference between both groups (67% vs. 54%, *p* = 0.259) (Table 3). However, multiple organ failure (25% vs. 7%, *p* = 0.019), and 30-day mortality (33% vs. 14%, *p* = 0.032) were higher in the CI group. A total of 23 (17%) patients died during the hospital stay, including 8 (33%) patients from the CI group and 15 (7%) from the non-CI group (*p* = 0.032). Causes of death included shock in nine (7%) patients, multiple organ failure in nine (7%) patients, sepsis in two (2%) patients, and cardiac insufficiency in three (2%) patients.

## 4. Discussion

The current study presents many surrogate markers of colonic ischemia after aortic RAAA, including norepinephrine dose > 64 µg/kg, operating time ≥ 200 min, and PH < 7.3. CI after RAAA is common, occurring most often in within the first 3 postoperative days. Patients with preoperative cardiopulmonary resuscitation or loss of consciousness had a significantly higher incidence of colonic ischemia. In addition, CI is more common after open abdominal aortic repair compared with endovascular therapy. The incidence of CI in patients after RAAA was 18%, which is comparable to former reports, with incidence ranging from 10.6% to 36% [4,5,17]. Moreover, demographic findings and the higher mortality rate among patients with CI are in line with previous reports [4,6]. However, in-hospital mortality among patients with CI in the current study is 33%, which is almost half of the mortality rate presented by a large German register evaluation (64.2%) [4].

Mean arterial pressure, heart rate, and daily fluid therapy were comparable in both CI and non-CI groups, revealing the sufficiency of the hemodynamic treatment. Appropriate fluid loading may help to avoid the use of high-dose catecholamines and may offer a protective factor against CI [18]. However, colloid and crystalloid resuscitation may carry many complications [19]. Although this study identified the same factors on univariate analysis compared to other studies [12], their statistical significance was not upheld in the multivariable models, where norepinephrine doses became more important than all other factors.

The current study showed that patients with colonic ischemia demonstrated a significant decrease in platelets during the first seven days after surgery. The reason for this may be the association with the excessive activation of the systemic inflammatory response reaction (SIRS), which, in turn, also manifests itself in an increased need for vasopressors. Although this is only a hypothesis supported by the comparable extent of fluid resuscitation in both groups excluding dilution effects. That said, we can only infer associations, not causation, from the data previously posted here.

There is a gap in the literature regarding the effect of vasoactive agents on colonic ischemia in humans. However, many animal studies have shown that catecholamines increase cardiac output and systemic oxygen delivery, but may decrease intestinal blood flow and oxygen delivery [20,21,22].

Although many studies have reported on CI after elective and emergency aortic surgery [7,9,11,23,24], only a few studies have addressed the hemodynamic variables associated with the development of CI after RAAA [6], and sometimes only reported it in subgroup analyses [12].

Therefore, the results of this work illuminate the importance of considering hemodynamic impairment, and especially catecholamine demand, as a possible surrogate parameter for the detection or prevention of CI after RAAA repair. This is further emphasized by our findings that all CI patients required catecholamine support, whereas only 70% of non-CI patients received any type of vasopressor or inotrope to maintain MAP.

Whereas re-implantation of the inferior mesenteric artery (IMA) may be considered in patients with elective repair of AAA, this procedure may be controversial in hemodynamically unstable patients who underwent emergency repair of RAAAs. Additionally, none of the previous studies found an increased risk of CI after IMA ligation [3,25,26]. Moreover, covering the IMA in EVAR patients did not increase the CI risk in many other studies [27,28,29,30].

The etiology associated with the occurrence of CI after RAAA is multifactorial [9]. One factor may be an exaggerated normal physiological response to maintain vital organ perfusion at the expense of mesenteric perfusion, as seen in non-occlusive mesenteric ischemia (NOMI) [31]. In addition, some studies have shown that preoperative shock and operative blood loss, both of which are associated with hemodynamic impairment, are the most critical predictors of colonic ischemia after RAAA repair [6,17].

Some of the previously reported risk factors, including preoperative resuscitation, preoperative hemodynamic instability, serum lactate, decreased pH (<7.3), the intraoperative need for more than five PRBC units, and open repair, are confirmed by the current study results [6,8]. Furthermore, CAD, preoperative loss of consciousness, and higher doses of norepinephrine were identified as additional predictors of CI in the present study. Whether to refer to MAP or SBP as well as the appropriate target blood pressure for maintaining adequate organ perfusion is still controversial, but there is some evidence that an SBP around 80 mmHg is associated with a higher rate of post-operative organ dysfunction. In addition, SBP can easily be measured and compared in the RAAA setting and is mostly a “faster” parameter than any blood (gas) analysis. Therefore, we chose a value <90 mmHg as the definition of hemodynamic instability in our analysis [32,33].

The answer to the question of whether norepinephrine dose is a cause, or a sign of CI is not clear in this study. However, there is an association between CI and norepinephrine dose escalation. Therefore, a more pronounced hemodynamic disturbance and an increased need for norepinephrine may increase the risk of CI. Conversely, CI with hemorrhage and sepsis may also increase the norepinephrine dose. Thus, the dose of norepinephrine administered can be interpreted as a predictor of poor outcomes. All measures should be taken to optimize the hemodynamic status of the patient; for example, increasing/ensuring adequate organ blood flow to the organ with as little vasopressor therapy as possible.

All patients requiring vasopressor therapy in this study used norepinephrine alone or rarely in combination with other catecholamines. Therefore, we analyzed and identified norepinephrine with a cutoff > 64 µg/kg for optimal risk prediction, rather than other catecholamines. Although animal studies did not show any changes in the mesenteric blood flow in norepinephrine-treated animals compared to placebo [34,35], the incidence of colonic ischemia showed a statistically significant relationship with the norepinephrine doses in the present study. Regarding the optimal choice for vasopressors, vasopressin analogs have been considered potentially useful vasopressors to maintain mesenteric perfusion compared to other catecholamines [36]. However, a randomized trial in patients with vasoplegic and septic shock did not demonstrate an advantage of a special agent in the occurrence of mesenteric ischemia [37].

Given this, it is unlikely that increased use of a catecholamines rather than norepinephrine would have led to different results. However, there is no literature on randomized clinical trials comparing different vasoactive agents in patients with acute mesenteric ischemia [36].

In addition, hemoglobin, the volume of administered fluids, MAP, and HR, all parameters important for hemodynamic stability, were within acceptable target ranges in intensive care and did not differ between groups.

Regarding the findings on the occurrence of CAD, Willemsen et al. and Behrendt et al. assessed the presence of a cardiac history without any differences between CI patients and non-CI patients [4,12]. In these studies, the cardiac history included the history of chronic heart failure (CHF), cardiac arrhythmias, antihypertensive medications, angina pectoris, diuretics, or digoxin, and having peripheral edema or cardiomegaly. However, the present study supports the premise that the presence of coronary artery disease should be assessed to predict the risk of CI, rather than a general summary of heart disease.

This study has a few limitations, including its single-institution retrospective design and the relatively small number of patients with colonic ischemia. The main limitation of our study is that it is an observational study in a cohort of patients with a ruptured aortic aneurysm, which may limit generalizability and lead to unmeasured confounding. Additionally, the sample size and the rarity of colonic ischemia may increase the risk of type II statistical errors. Moreover, the exact number of patients with colonic ischemia stage I is unknown because not all patients undergo routine endoscopy. In addition, colonic ischemia can be difficult to discern intraoperatively by looking at the outside of the colon. Furthermore, the extent of ischemia is difficult to determine. Therefore, we recommend colonoscopy before or during surgery as a standard of care. In addition, advanced hemodynamic parameters such as stroke volume measurement or echocardiography, which provide additional information on the appropriate choice of catecholamines on a case-by-case basis, were not routinely performed during intensive care and, therefore, could not be evaluated in this retrospective work.

## 5. Conclusions

Colonic ischemia after ruptured aortic aneurysms is a multifactorial complication. Our results highlight the potential role of vasopressors as a surrogate parameter for the development of CI after RAAA surgery.

## Figures and Tables

**Table 1:** Risk factors and descriptive characteristics of the study population.

Characteristics	Total	CI	Non-CI	OR (95% Confidence Interval)	Univariate Analysis (*p*)
Number	135 (100)	24 (18)	111 (82)		
Demographics					
Age (y)	75 (IQR 68–81)	79 (IQR 72–81)	74 (IQR 67–81)	0.97 (0.92–1.02)	0.220
Male	111 (82)	22 (92)	89 (80)	2.7 (0.6–12.4)	0.246
Medical history					
Coronary artery disease	28 (21)	9 (38)	19 (17)	2.9 (1.1–7.6)	0.026 *
Diabetes mellitus	25 (19)	6 (25)	19 (17)	1.6 (0.6–4.6)	0.389
Hypertension	103 (76)	19 (79)	84 (76)	1.2 (0.4–3.6)	0.715
Hyperlipoproteinemia	19 (14)	2 (8)	17 (15)	0.5 (0.1–2.3)	0.525
COPD	34 (25)	5 (21)	29 (26)	0.7 (0.3–2.2)	0.588
Renal insufficiency	46 (34)	9 (38)	37 (33)	1.2 (0.5 –3.0)	0.696
Smoking	41 (30)	9 (38)	32 (29)	1.5 (0.6–3.7)	0.402
Peripheral arterial disease	15 (11)	4 (17)	11 (10)	1.8 (0.5–6.3)	0.307
Malignancy	22 (16)	4 (17)	18 (16)	1.0 (0.3–3.4)	1
Preoperative status of IMA					
Patent	68 (50)	8 (33)	60 (54)	2.4 (0.9–5.9)	0.066
Occluded	67 (50)	16 (67)	51 (46)	2.4 (0.9–5.9)	0.066
Postoperative status of patent IMA					
IMA ligation	37 (27)	6 (25)	31 (28)	0.5 (0.1–1.9)	0.406
IMA replantation	3 (2)	1 (4)	2 (2)	2.4 (0.2–27.2)	0.447
IMA Overstenting	28 (21)	1 (4)	27 (24)	0.1 (0.02–1.1)	0.045
Status of internal iliac arteries					
Patent	116 (86)	18 (75)	98 (88)	0.4 (0.1–1.2)	0.107
Unilateral IIA occlusion	11 (8)	3 (13)	8 (7)	1.8 (0.5–7.5)	0.412
Bilateral IIA occlusion	8 (6)	3 (13)	5 (5)	3.0 (0.7–13.7)	0.150
Uni-/Bilateral IIA occlusion	19 (14)	6 (25)	13 (12)	2.5 (0.8–7.5)	0.107

CI: Colonic ischemia, OR: Odds ratio, IMA: inferior mesenteric artery, IIA: internal iliac artery. The asterisk indicates statistical significance.

**Table 2:** Univariate logistic regression of the preoperative, intraoperative, and first postoperative (POD1) risk factors for colonic ischemia. Multivariable analysis and risk difference of the obtained predictors.

Characteristics	Total	CI	Non-CI	OR (95% Confidence Interval)	*p*
Number	135 (100)	24 (18)	111 (82)		
Univariate analysis					
Preoperative Variables					
Coronary artery disease	28 (21)	9 (38)	19 (17)	2.9 (1.1–7.6)	0.026 *
Preoperative hemodynamic instability	54 (40)	17 (71)	37 (33)	4.9 (1.9–12.7)	<0.001 *
Preoperative cardiopulmonary resuscitation	11 (8)	6 (25)	5 (5)	7.0 (2.0–25.6)	0.004 *
Preoperative loss of consciousness	27 (20)	10 (42)	17 (15)	3.9 (1.5–10.3)	0.009 *
Age > 76 y	61 (46)	14 (58)	47 (43)	1.8 (0.8–4.5)	0.153
Maximum aortic diameter in mm	78 ± 21	85 ± 16	77 ± 21	0.98 (0.96–1.0)	0.129
Intraoperative Variables					
Open aortic repair	98 (73)	22 (92)	76 (69)	5.1 (1.1–22.7)	0.021 *
PRBCs ≥ 5 units	71 (53)	18 (75)	53 (48)	3.3 (1.2–8.9)	0.015 *
Suprarenal clamping	32 (24)	6 (25)	26 (23)	1.1 (0.4–3.0)	0.869
Operating time ≥ 200 min	67 (50)	18 (75)	49 (44)	3.8 (1.4–10.3)	0.006 *
Variables from POD1					
NE > 64 µg/kg	45 (33)	20 (83)	25 (23)	17.2 (5.4–55.0)	<0.001 *
PH < 7.3	56 (42)	17 (71)	39 (35)	4.5 (1.7–11.7)	0.001 *
arterial lactate > 5 mmol/L	49 (36)	19 (79)	30 (27)	10.3 (3.5–29.9)	<0.001 *
MAP	79 ± 9	79 ± 8	79 ± 9	0.99 (0.95–1.05)	0.977
MAP < 65 mmHg	10 (7)	2 (8)	8 (7)	1.17 (0.23–5.89)	1.0
HR	85 ± 14	87 ± 14	85 ± 15	0.99 (0.96–1.02)	0.495
Hemoglobin < 9 g/dL	27 (20)	8 (33)	19 (17)	2.4 (0.9–6.3)	0.091
FT ≥ 5 L	40 (30)	10 (42)	30 (28)	1.9 (0.8–4.7)	0.154
Creatinine > 190 mmol	12 (9)	3 (13)	9 (8)	1.6 (0.4–6.4)	0.447
Multivariable analysis				RD (95% confidence interval)	
NE > 64 µg/kg				0.40 (0.25–0.55)	<0.001 *
Operating time ≥ 200 min				0.18 (0.05–0.31)	0.042 *
PH < 7.3				0.21 (0.07–0.35)	0.019 *

CI: colonic ischemia; POD1: postoperative day one; OR: Odds ratio; RD: risk difference; PRBC: packed red blood cells; FT ≥ 5 L: daily fluid therapy more than 5 L; NE: Norepinephrine; MAP: mean arterial pressure; the asterisk indicates statistical significance.

**Table 3:** Postoperative complications and outcomes in CI and non-CI patients after RAAA.

	Total	CI	Non-CI	*p*
**Complications**				
Overall	76 (56)	16 (67)	60 (54)	0.259
Respiratory	59 (44)	10 (42)	49 (44)	0.824
Cardiac	26 (19)	7 (29)	19 (17)	0.251
Abdominal compartment syndrome	10 (7)	4 (17)	6 (5)	0.077
Renal failure	40 (30)	10 (42)	30 (27)	0.154
Multiple organ failure	14 (10)	6 (25)	8 (7)	0.019 *
Sepsis	18 (13)	4 (17)	14 (13)	0.527
**Outcome**				
ICU length of stay (days)	7 (IQR 3–16)	15 (IQR 6–16)	6 (IQR 2–15)	0.018 *
Hospital length of stay (days)	12 (IQR 9–22)	21 (IQR 10–23)	12 (IQR 9–20)	0.174
30-day mortality	23 (17)	8 (33)	15 (14)	0.032 *

The asterisk indicates statistical significance.

## Data Availability

The data presented in this study are available on request from the corresponding author.
